# Engineering Carbons by Elemental Doping and Processing Procedures for Design of Functional Materials

**DOI:** 10.1002/smll.202503508

**Published:** 2025-07-16

**Authors:** Yue Shao, Kanglei Pang, Hong Wang, Jiayin Yuan

**Affiliations:** ^1^ Key Laboratory of Functional Polymer Materials of the Ministry of Education Institute of Polymer Chemistry College of Chemistry Nankai University Tianjin 300071 P. R. China; ^2^ Wallenberg Initiative Materials Science for Sustainability Department of Chemistry Stockholm University Stockholm 10691 Sweden

**Keywords:** carbon materials, electrocatalysts, heteroatoms doping, heterogenous catalysis, metal free

## Abstract

Carbon materials have emerged as versatile and promising candidates due to their low cost, abundance, and exceptional thermal and chemical stability. Doping has proven to be a powerful strategy for further enhancing their properties and expanding their application scope. Nonetheless, challenges remain in achieving performance comparable to established materials such as noble metal or metal oxide catalysts, gaining molecular insights into the underlying mechanisms, and ensuring controllable synthesis. This minireview explores the next frontier in the field by discussing less commonly studied doping elements, such as halogens (fluorine) and semi‐metallic elements. The unique features of these elements in carbon are specifically debated, including their configurations, structural properties, and chemical behavior, highlighting their differences compared to other heteroatoms. Additionally, the synergistic effects among different dopants within the carbon matrix are highlighted, particularly through the emerging concept of solid‐state Frustrated Lewis Pairs (FLP) in various applications. Furthermore, the critical role of carbonization precursors and techniques in the design of advanced carbon materials is emphasized, focusing on the relationships between processes and properties. Through exploring these emerging avenues, the development of next‐generation carbon materials is anticipated with enhanced functionalities and performance.

## Graphical abstract

This mini‐review explores the emerging role of less‐studied elements doping in engineered carbon materials. It highlights their unique electronic effects, synergistic co‐doping strategies, and importance of processing conditions. These insights offer new directions for developing advanced carbon‐based materials with enhanced performance in catalysis, energy storage, and environmental applications.

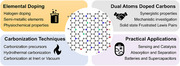

## 1 Introduction

In modern science and technology, carbon materials play a unique role and have been widely utilized across fields, ranging from catalysis, adsorption/separation, to energy conversion and storage.^[^ ^1^ ^]^ Their unusually broad scope of applications is associated with their exceptional physicochemical properties addressable in a wide range, e.g., surface area and porosity, electrical and thermal conductivity, chemical stability, and apparent density. One of the latest advance in carbon research has been made by engineering carbon with heteroatoms as dopant, e.g., covalently bonding nitrogen (N) into a carbon framework, to widen their property and application spectrum.^[^ ^2^ ^]^ For example, N‐doped carbon has been shown to serve as a better support for Pt catalyst than commercial activated carbon in many cases, where N atoms on carbon surface stabilizes Pt nanoparticles more efficiently, and N atoms in the bulk carbon modifies the electronic structure and thus its work‐function to align the Pt/carbon Schottky junction for catalysis.^[^ ^3^ ^]^

In 2009, N‐doped carbon materials in the form of nanotubes were reported to exhibit remarkable reactivity in electrocatalytic oxygen reduction comparable to noble metal catalyst (Pt/C), which spurred significant interest in heteroatom‐doped carbon materials.^[^ ^4^ ^]^ The enhanced reactivity was assigned to different electronegativity between N and C atoms. Theoretical studies suggest that in N‐doped carbons, the binding energy of O₂ is stronger due to the overall positive charge induced on the carbon atoms adjacent to the N one. This alteration in charge distribution changes the O₂ adsorption mode from an end‐on to a side‐on configuration. This seemingly subtle shift is significant enough to produce a reactivity level in N‐doped carbons that is fairly comparable to precious metals, fueling the development of cost‐effective and efficient metal‐free catalysts. A pyramid of heteroatoms, semi‐metallic elements, and halogens has been deliberately incorporated into various carbon allotropes, including graphene (single or few‐layer sheets with up to 10 layers, featuring Dirac points and zero‐bandgap semimetal behavior), graphite (stacked graphene layers with more than 10 layers, exhibiting interlayer interactions and modified band structures), turbostratic carbon (with disordered, rotationally misaligned layers, leading to reduced crystallinity and enhanced edge/defect doping), carbon nanotubes, and amorphous carbons, to investigate and further optimize their performance. These allotropes influence doping outcomes differently; for instance, doping single‐layer graphene shifts the Fermi level due to its Dirac points, while graphite's interlayer interactions and turbostratic carbon's disordered structure favor dopant incorporation at basal planes or edge sites, respectively. This incorporation has significantly expanded their applications beyond energy‐related uses to include sensing, biomedicine, and as composites absorbents for electromagnetic radiation, among others. The critical roles of heteroatoms in these applications are substantial and have been extensively reviewed, particularly regarding how they enhance the electronic properties, chemical reactivity, and overall performance of carbon‐based materials. It's worth noting that introducing foreign elements to bond the carbon matrix can cause structural defects, leading to lattice distortion.^[^ ^5^ ^]^ This process, known as defect engineering, has recently been demonstrated effective for regulating the electronic structure of carbon materials, further enhancing their potential for diverse technological applications.^[^ ^6^ ^]^ These heteroatoms “turned” carbon‐based materials a value‐added metal‐free alternative to traditional metals or metal oxides in many catalytic systems, offering numerous advantages, e.g., low‐cost, large surface areas, absence of metal, chemical stability, and tuneable conductivity and electrochemical activity.^[^ ^7^ ^]^ In addition, beyond heteroatom doping, constructing heterointerfaces between heteroatom‐doped carbon and other functional phases such as transition metal dichalcogenides, MXenes, and metal oxides yields further synergy.^[^ ^8^ ^]^ These heterointerfaces can be classified based on their interaction type—van der Waals or covalent—and their structural arrangement, either vertical (stacked layers) or lateral (in‐plane). Each configuration offers distinct advantages, driven by the chemistry at the heterointerface, which influences electronic band alignment, charge transfer, and surface properties.^[^ ^9^ ^]^ By tailoring doping types and optimizing heterointerface chemistry, these materials can achieve superior functionality across diverse applications.

Looking into the future, the question arises, what is the next step for heteroatom‐doped carbon materials? While there have been numerous excellent reviews focusing on heteroatom‐doped carbons and their applications, we aim to highlight less‐studied but rather crucial aspects of these materials.^[^ ^10^ ^]^ In this minireview, we will first discuss heteroatoms that are not commonly investigated, especially compared to conventional heteroatoms (i.e., N), such as halogens and semi‐metallic elements. These dopants exhibit distinctive properties and have promising applications in various fields that have yet to be fully explored through conventional doping methods. For instance, halogens can facilitate surface modification, and their configuration is distinctively different from conventional heteroatoms. Semi‐metallic elements can impart unique metallic characteristics that are analog to single atom catalysis, while both structurally and chemically different. It is worth highlighting the synergistic effects of individual dopants in the carbon matrix, an area that has garnered increasing attention. Notably, the concept of solid‐state frustrated Lewis pairs, which involves the co‐doping of electron‐deficient and electron‐rich elements, has demonstrated a dramatic increase in performance compared to their single‐doping counterparts. Finally, we will explore the importance of carbonization precursors and carbonization craft for designing carbon materials. These aspects are equally important for the development of innovative carbon materials but have been rarely debated in the literature. Our goal here is to challenge our current knowledge and draw attention to this emerging area so to push forward the research boundaries of carbon materials.

## 2 Halogen (Fluorine) Doped Carbons

The significant electronegativity difference of (N, 3.04) and (C, 2.55), and the electron‐rich nature of N atom, both can grow structural heterogeneity in the carbon matrix and thus deviate the electronic structures from traditional carbon materials. By contrast, halogen‐doped carbons have generally been less studied, presumably due to challenges associated with their preparation and structural characterization. The fact is such carbon materials have demonstrated intriguing activity in catalytic systems, despite limited understanding of their mechanisms, suggesting untapped potential applications worth exploration.

**Figure 1:**
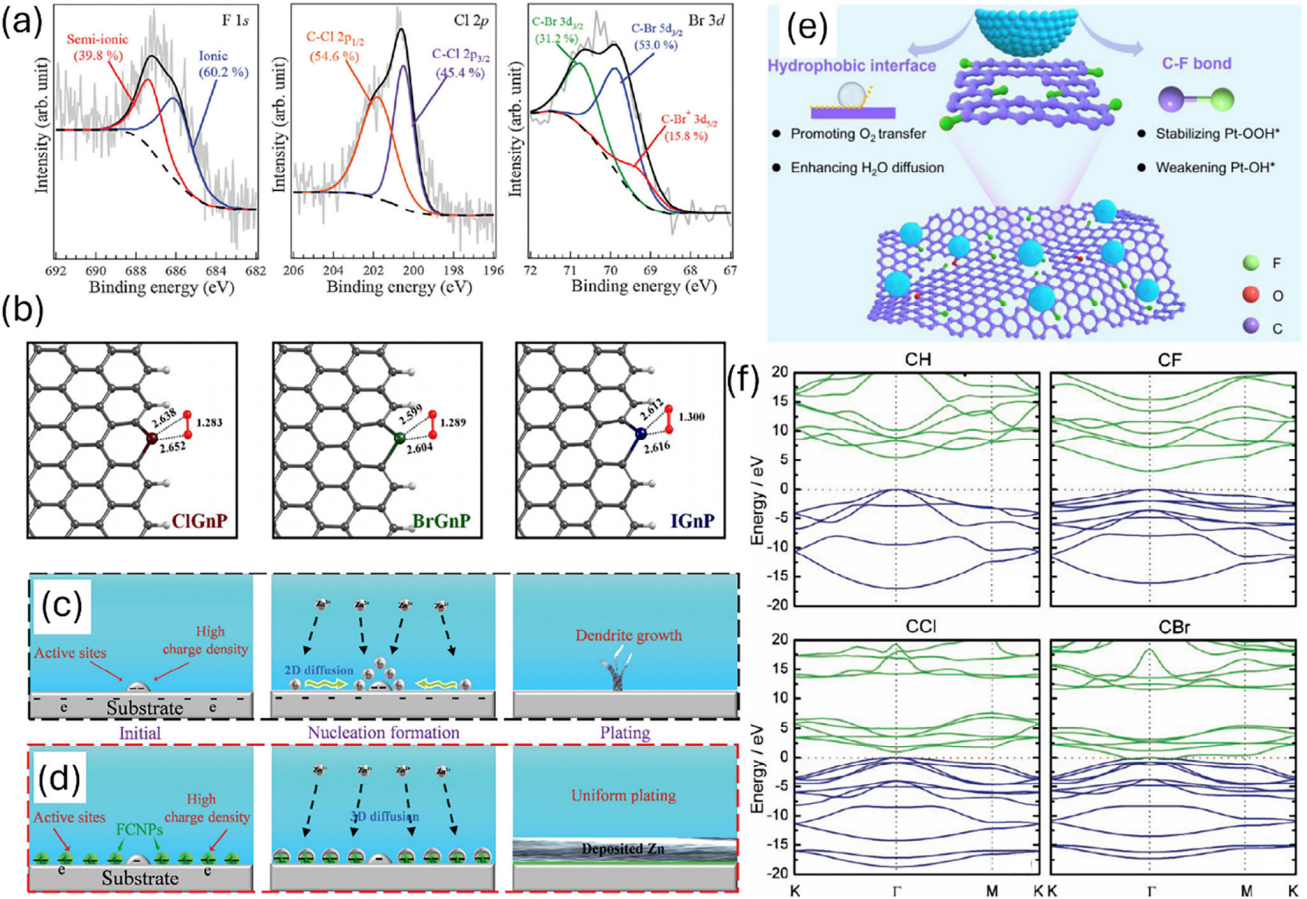
a) High resolution XPS spectra of F 1s in F doped carbons, Cl 2p in Cl doped carbons, and Br 3d in Br doped carbons. Reproduced with permission.^[^ ^14^ ^]^ Copyright 2016, Royal Society of Chemistry. b) The optimized O₂ adsorption geometries on exfoliated graphene nanoplatelets, where each halogen atom is covalently linked to two sp^2^ carbon atoms. Reproduced with permission.^[^ ^17^ ^]^ Copyright 2013, Nature Publishing Group. Schematic illustration of zinc plating behavior on different substrates: c) depicts the behavior on a bare metal substrate, while (d) shows the effect of CF₄ plasma treatment. Reproduced with permission.^[^ ^21^ ^]^ Copyright 2023, Wiley‐VCH. e) Schematic illustration of the promoting effect of hydrophobic and polar C─F bond on ORR. The blue sphere indicates the water droplet. Reproduced with permission.^[^ ^22^ ^]^ Copyright 2024, Wiley‐VCH. f) The electronic band structure near the bandgaps of various halogenated carbon compounds, including C─H, C─F, C─Cl, and C─Br, along the high‐symmetry lines (K, Γ, M) in the Brillouin zone. The zero‐energy level is set to the valence band maximum. Reproduced with permission.^[^ ^24^ ^]^ Copyright 2010, Wiley‐VCH.

One of the notable features of halogens is their high electronegativity, i.e., 3.98 for fluorine (F), 3.16 for chlorine (Cl), 2.96 for bromine (Br), and 2.66 for iodine (I). They can polarize adjacent carbon atoms, imparting them with a partially positive charge. As a result, doping of halogens is prone to increase the hole concentration and shifts the Fermi level below the Dirac point, which results in p‐type doping, where holes are the primary charge carriers.^[^ ^11^ ^]^ In a sharp contrast, heteroatoms such as N or S exhibits extra valence electrons are effective for n‐type doping. The positive charge characteristics on the adjacent carbon atoms can also be induced by the large size of halogens, and induces heterogeneity onto the carbon lattice, resulting in asymmetric spin and charge distribution. These aspects are usually responsible for their activity.^[^ ^12^ ^]^ For example, introducing F atoms can create Lewis acid sites due to the significant mismatch in electronegativity between F and C, which was reported to boost electroreduction of NH_3_.^[^ ^13^ ^]^ The yield rate of NH_3_ reached 197.7 µgNH_3_ mg¯¹cat. h¯¹, which is more than one order of magnitude higher than the state‐of‐the‐art metal‐free catalysts under similar conditions. The Lewis acid sites, created by F doping, can repel protons (H) and lead to a repulsion between the Lewis acid site and the proton, thus inhibiting the HER and promoting the selective conversion of N_2_ to NH_3_. Note, unlike N doped carbons, wherein the ORR activity typically follows the electronegativity of the doping element, halogen‐doped carbons do not necessarily correlate their ORR activity directly with the electronegativity of the halogen atoms.^[^ ^14^ ^]^ This apparent inconsistency between halogens and conventional heteroatoms may be attributed to the varied intrinsic bonding modes. In halogen‐doped carbons, F, Br, and I can form partially ionized bonds with carbon atoms, whereas Cl tends to form dominantly covalent bonds (**Figure** **1**a). The significant ionic character of these bonds plays a crucial role in enhancing reactivity. These bonds can act bifunctionally as electron acceptors or donors, facilitating charge transfer between the halogen and carbon atoms. In a direct comparison, the electrochemical performance of highly fluorinated graphite was evaluated by synthesizing materials dominated by either covalent or semi‐ionic C─F bonds. X‐ray photoelectron spectroscopy (XPS) analysis revealed distinct binding energies: covalent C─F bonds exhibit a C_1s_ peak at ∼291.8 eV, indicative of strong, less polar bonds, while semi‐ionic C─F bonds show a peak at ∼290.2 eV, suggesting partial electron transfer from carbon to fluorine, creating polarized, Lewis acid‐like sites.^[^ ^15^ ^]^ This polarization enhances the reactivity of semi‐ionic C─F bonds. The study demonstrated that fluorinated carbons with predominantly semi‐ionic C─F bonds, prepared using KAgF₄ and elemental fluorine, achieved high discharge potentials (3.2 V vs Li/Li⁺) and capacities (400–450 mA h g⁻¹) in primary lithium batteries, significantly outperforming those with covalent C─F bonds (200–370 mA h g⁻¹).^[^ ^16^ ^]^ The superior performance is attributed to the semi‐ionic bonds’ higher activity and fluorine diffusibility within conductive, planar graphene layers. Subsequent research further highlighted the catalytic potential of semi‐ionic C─F bonds in fluorine‐doped carbon catalysts for the ORR. These catalysts exhibited remarkable efficiency, with a peak potential of ‐0.19 V (vs Ag/AgCl), surpassing that of commercial Pt/C catalysts. High‐resolution XPS spectra confirmed a semi‐ionic C─F bond peak at ∼289.8 eV. The polarized sites and maintained conductivity of semi‐ionic C─F bonds enable efficient electron transfer and reactant activation, making fluorinated carbons with these bonds promising for both electrochemical and electrocatalytic applications. These distinctive bonding behaviors contribute to the unique electronic properties and reactivity patterns seen in halogen‐doped carbon materials, which is different from conventional heteroatom doping. In addition, the atomic sizes of Br and I are drastically larger than that of Cl. Hence the valence electrons of Br and I are much loosely bound to carbon than those of Cl for facilitating charge polarization (Figure 1b).^[^ ^17^ ^]^ On the other hand, the smaller F can indeed fully cover the graphene sheet to form fluorographene (CF)n by fluorinating graphene using XeF_2_ at various temperatures under an inert atmosphere.^[^ ^18^ ^]^ Unlike other types of halographene, this is the only stable form for fluorine doping, and (CF)n acts as an insulator (≈10⁻⁴ S m^−1^) by restricting the delocalized electrons, while partially fluorinated graphene with semi‐ionic C─F bonds retains moderate conductivity (≈10⁴ S m^−1^).^[^ ^19^ ^]^

Surface modification is another crucial aspect of fluorine (F) doping. For example, due to the strong hydrogen bonding capability, F atoms can help stabilize, via hydrogen bond, the surface structure and prevent photobleaching.^[^ ^20^ ^]^ In F‐doped carbon dots, it contributes to aggregation and piezochromic behavior, where fluorescence intensity increases with pressure, indicating that aggregation enhances emission. F atoms were also reported to induce high surface charge density, acting as nucleation assistors that form numerous homogeneous Zn nucleation sites for 3D growth (Figure 1c,d).^[^ ^21^ ^]^ This improved uniform Zn plating on electrode, and enabled the cell to operate for 100 h at a high current density of 50 mA cm⁻^2^, underscoring the excellent rate capability of F‐doped carbon‐supported Zn electrodes. Surface chemistry determined to a large extent the catalytic performance of catalysts. Incorporating F atoms creates C─F bonds, which weakens the binding energy of the *OH intermediate on Pt catalyst surfaces, while affecting less binding energy of *OOH. Additionally, it can create a local super‐hydrophobic interface on the catalyst surface, facilitating the diffusion of H₂O and mass transfer of O₂ for enhanced ORR kinetics (Figure 1e).^[^ ^22^ ^]^ This strategy addresses limitations imposed by the scaling relationship and optimizes the interfacial microenvironment for mass transport to perform ORR better. Similar results have been reported in recent studies, highlighting the impact of interfacial microenvironments of halogen doping on the activity and performance of carbon electrocatalysts.^[^ ^23^ ^]^

It is also worth noting that different halogens vary the bandgap of carbon materials differently.^[^ ^24^ ^]^ Pristine graphene is a zero‐bandgap semimetal with high electrical conductivity (≈10^6^ S m^−1^) due to its sp^2^‐hybridized carbon lattice and π‐conjugated electron cloud. However, introduction of halogen atoms can convert sp^2^ to sp^3^ hybridization, disrupting the π‐system, and opening a bandgap, which reduces conductivity of the resulting carbons. Theoretical calculations show that graphene fluoride has the largest bandgap of 3.1 eV, while 0.9 eV for Cl‐doped one and almost zero for Br‐doped one (Figure 1f). However, codoping of N and F leads to exceptionally low bandgap of 0.77 eV, which is significantly lower than F (1.556 eV) and N (1.584 eV) doped carbons. It highlights the power of halogen atoms to tune the electronic properties of 2D materials by altering their chemical composition.

In a short conclusion, one of the most important aspects of halogen‐doped carbons is their configuration within the carbon matrix. For F‐doped carbons, the C─F bonds can be partially ionic and vary its ionic contribution by carbon's hybridization state, distinguishing them from conventional heteroatom‐doped carbons, e.g. N with a similar electronegativity to carbon.^[^ ^25^ ^]^ Notably, partially ionic C─F bonds exhibit exceptionally high polarities, resulting in a significant positive charge localized on the adjacent carbon atoms.^[^ ^26^ ^]^ Additionally, F atoms tend to position themselves at the edges of the carbon structure. Unlike larger heteroatoms such as S and P, edge C─F bonds effectively repel substances like water and O₂ from neighboring carbon atoms due to their large electronegativity difference, thereby significantly enhancing their resistance to environmental corrosion during their applications. Furthermore, a remarkable synergy between F atoms and other dopants, particularly nitrogen (N) atoms, has been observed, leading to maximum charge polarization and spin density distribution. This interaction generates highly active catalytic sites, surpassing the catalytic performance of N‐doping alone or other co‐doping systems, such as N‐S or N‐P.^[^ ^27^ ^]^ The unique properties and synergistic effects of halogen‐doped carbon materials underscore their potential for advanced applications in various fields.

## 3 Dual Atoms Doped Carbons

Doping a carbon framework with two or more elements can induce synergistic effects between the heteroatoms, attracting significant attention, particularly combinations like P/N, B/N, and S/N.^[^ ^28^ ^]^ Notably, N/F co‐doped carbons exhibit remarkable reactivity, outperforming their individual single‐doped carbons. This dramatic enhancement is attributed to their optimized charge polarization and spin density distribution, resulting in the formation of highly active catalytic sites. Various examples of co‐doping in carbon materials exist, with mechanisms of enhancement varying based on the specific dopants involved. However, synthesizing heteroatom‐doped carbons with an exclusive sole configuration remains a challenge, which can hinder our understanding of their enhanced performance. In this mini‐review, we emphasize a unique form of dual doping known as solid‐state “Frustrated Lewis Pairs” (FLP) chemistry. This concept has recently been applied to explain the enhanced catalytic performance observed in solid‐state heterogeneous systems, offering new insights into the design and functionality of advanced catalytic materials.

**Figure 2:**
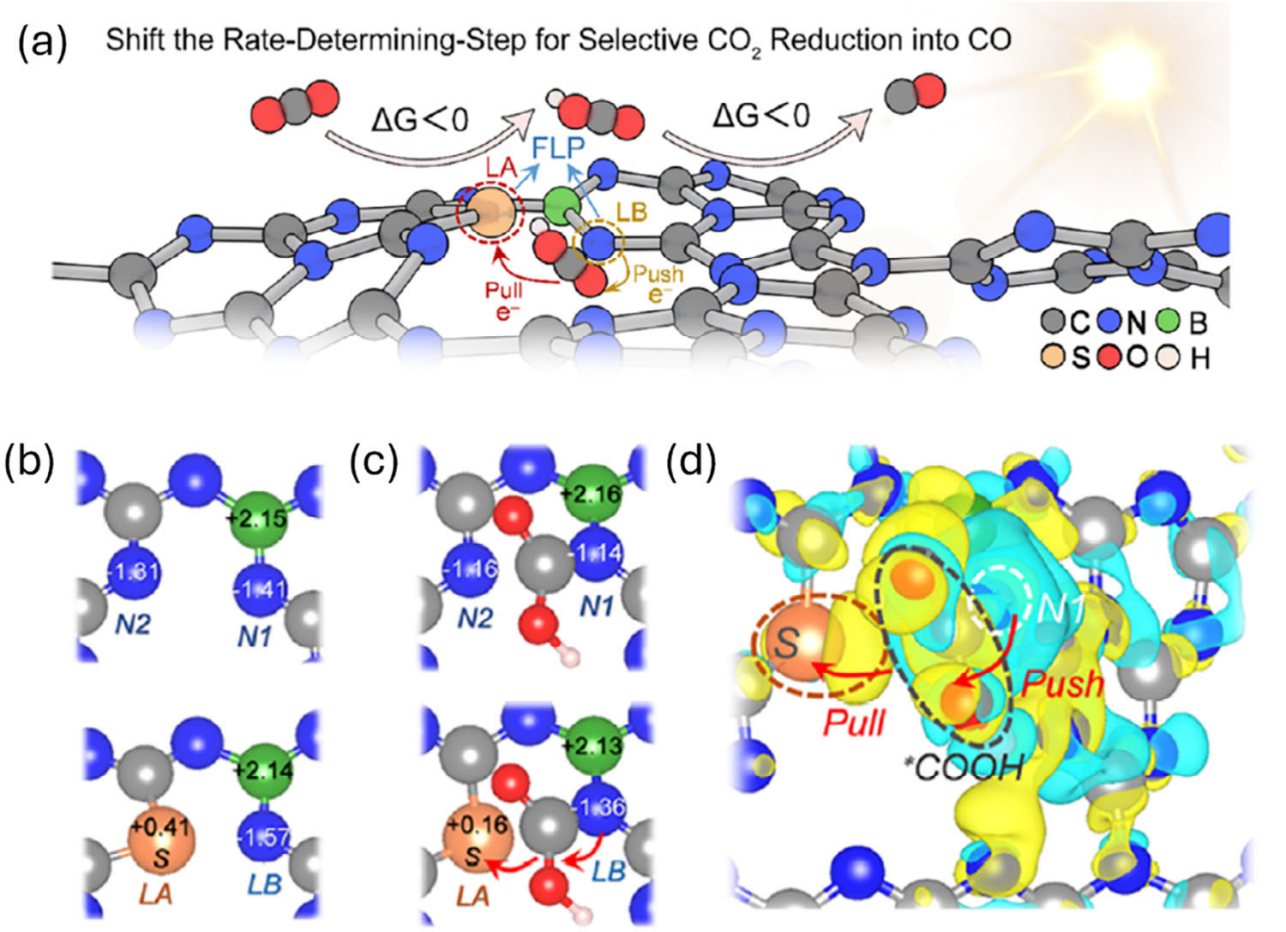
a) Illustration of the photocatalytic reduction of CO₂ to CO using B,S‐carbon nitride. b) Calculated Bader charges for B‐carbon nitride and B,S‐carbon nitride at different atomic sites. c) Bader charges for *COOH‐adsorbed B‐CN and *COOH‐adsorbed B,S‐ carbon nitride. d) Charge density difference of *COOH adsorbed on B,S‐ carbon nitride. The different colored spheres represent: blue for nitrogen (N), grey for carbon (C), green for boron (B), orange for sulfur (S), red for oxygen (O), and pink for hydrogen (H) atoms. Reproduced with permission.^[^ ^37a^ ^]^ Copyright 2023, American Chemical Society.

FLP chemistry was originally proposed in solution state reactions.^[^ ^29^ ^]^ Erker and colleagues showed that the solid‐state FLP comprising PCy_3_ and B(C_6_F_5_)_3_ could split H_2_ and trap SO_2_.^[^ ^30^ ^]^ Experimental and computational analysis suggests that the reaction takes place on the surface interface between particles of the phosphine and borane, subsequently behaving as a “molten” material, and ultimately affording a homogeneous solid sample of the phosphonium hydroborate salt. The above examples suggest that fully heterogeneous FLP catalysis is possible, and different heterogenous catalysts have been proposed.^[^ ^31^ ^]^ Carbon materials can be simultaneously doped with electron‐rich elements (e.g., N) and electron‐deficient elements (e.g., B). These dual dopants create immobilized Lewis acid and base sites on the graphitic carbon, wherein B atom is intrinsically electron‐deficient and N atom is electron‐rich.^[^ ^32^ ^]^ N 1S X‐ray photoelectron spectroscopy (XPS) revealed N atoms being incorporated into the graphene lattice chiefly as “pyridinic” and “graphitic” configurations, as well as forming N─B bonds. These B, N co‐dopants enhanced selectivity and reactivity toward the oxidation of benzylamine. The presence of B atoms (as electron acceptors) and N atoms (as electron donors) can, in principle, lower down the valence band (or HOMO) and elevate the conduction band (or LUMO). It expands the bandgap and induces electron relocalization, thus activating the substrate and O_2_ for oxidation reactions. This synergy resembles some homogeneous catalytic systems, particularly in FLPs chemistry. FLPs, first discovered by Stephan et al., involve sterically hindered Lewis acid‐base pairs that can reversibly activate H₂.^[^ ^29^ ^]^ In solid‐state chemistry, FLP‐like configurations have also successfully catalyzed various reactions, such as heterogeneous catalytic hydrogen of CO₂.^[^ ^33^ ^]^ Notably, B,N co‐dopants render carbon a similar FLP‐like reactivity toward H₂.^[^ ^34^ ^]^ According to theoretical calculations the H─H bond is cleaved at the B site, and one H atom was transferred to a N atom to form a stable product.^[^ ^35^ ^]^ In subsequent study, B,N‐doped carbons were observed to photocatalytically reduce CO₂ to multicarbon products.^[^ ^36^ ^]^ DFT calculations suggested that B,N‐doped carbons bind CO₂ more strongly in a bidentate configuration involving N−C and B−O interactions compared to a monodentate N−C structure. This synergistic effect within the carbon matrix is often referred to as a “push‐pull” effect in FLP configurations (**Figure** **2**a).^[^ ^37^ ^]^ That is, the N atom “pushes” electrons to *CO₂, facilitating its protonation into *COOH, while a sulfur atom “pulls” electrons to some extent from *COOH (Figure 2b,c). This “push‐pull” behavior grants *COOH an optimal adsorption energy, crucial for the spontaneous formation of *COOH and *CO intermediates (Figure 2d). In a recent study, B, N co‐doped FLPs catalysts were prepared and applied in the selective hydrogenation of α,β‐unsaturated aldehydes to unsaturated alcohols.^[^ ^38^ ^]^ In situ CO‐FTIR spectroscopy reveals that the catalyst preferentially adsorbs the C═O group of cinnamaldehyde in a vertical configuration, facilitated by the Lewis acidic B–N sites, while the Lewis basic pyridinic N sites activate H₂ via heterolytic cleavage. This “push‐pull” mechanism lowers the energy barrier for C═O hydrogenation while suppressing C═C bond reduction, which is thermodynamically favored (578.8 kJ mol^−1^ vs 705.8 kJ mol^−1^ for C═O). DFT calculations further support this mechanism, showing a low activation energy of 0.21 eV for H₂ dissociation at the FLP sites and a favorable adsorption energy of −1.45 eV for cinnamaldehyde's C═O group. The electron localization function and partial density of states analyses indicate strong electronic coupling between the B–N pairs and the carbon framework, enhancing charge transfer to the adsorbed substrate. The study also explores the hydrogenation pathways using transition state calculations, identifying transition state 1 (C═O hydrogenation) as the rate‐determining step with an energy barrier of 0.65 eV, significantly lower than that for C═C hydrogenation (1.12 eV). These findings highlight the FLP sites’ ability to modulate selectivity by stabilizing the transition state for C═O reduction.

Heterogeneous FLP catalysis, compared to the homogeneous one, is still in its infancy. However, carbon‐based FLPs have shown promising perspective for their excellent thermal and chemical stability. Graphitic and aromatic backbones resist oxidative degradation, and the spatial isolation of Lewis acid and Lewis base reduces deactivation via quenching. They are easily separable and recyclable, retaining activity across multiple cycles, which is crucial for industrial processes. In addition, their porous carbon frameworks also provide accessible active sites and facilitate substrate diffusion. The development of industrial dual heteroatoms‐doped carbon heterogeneous catalysts utilizing FLP chemistry and deeply understand underlying catalytic mechanism present an intriguing and important area of research.

## 4 Atomically Dispersed Semi‐Metallic Elements

Single atom catalysts (SACs) represent the cutting edge in catalyst design, offering maximum exposure of active sites and thereby maximizing catalytic efficiency in various chemical reactions.^[^ ^39^ ^]^ Carbon‐supported SACs are particularly interesting due to their unique physicochemical properties, and beneficial synergy between active catalytic sites and carbon substrates.^[^ ^40^ ^]^ Typically, SACs are anchored on a carbon matrix through strong electron‐donating elements to form stable metal‐ligand configurations, such as M‐N_4_. Alternatively, they can be loaded into specific defect sites within engineered carbons. Despite promises, the stability of SACs remains a significant long‐term challenge.^[^ ^41^ ^]^

**Figure 3:**
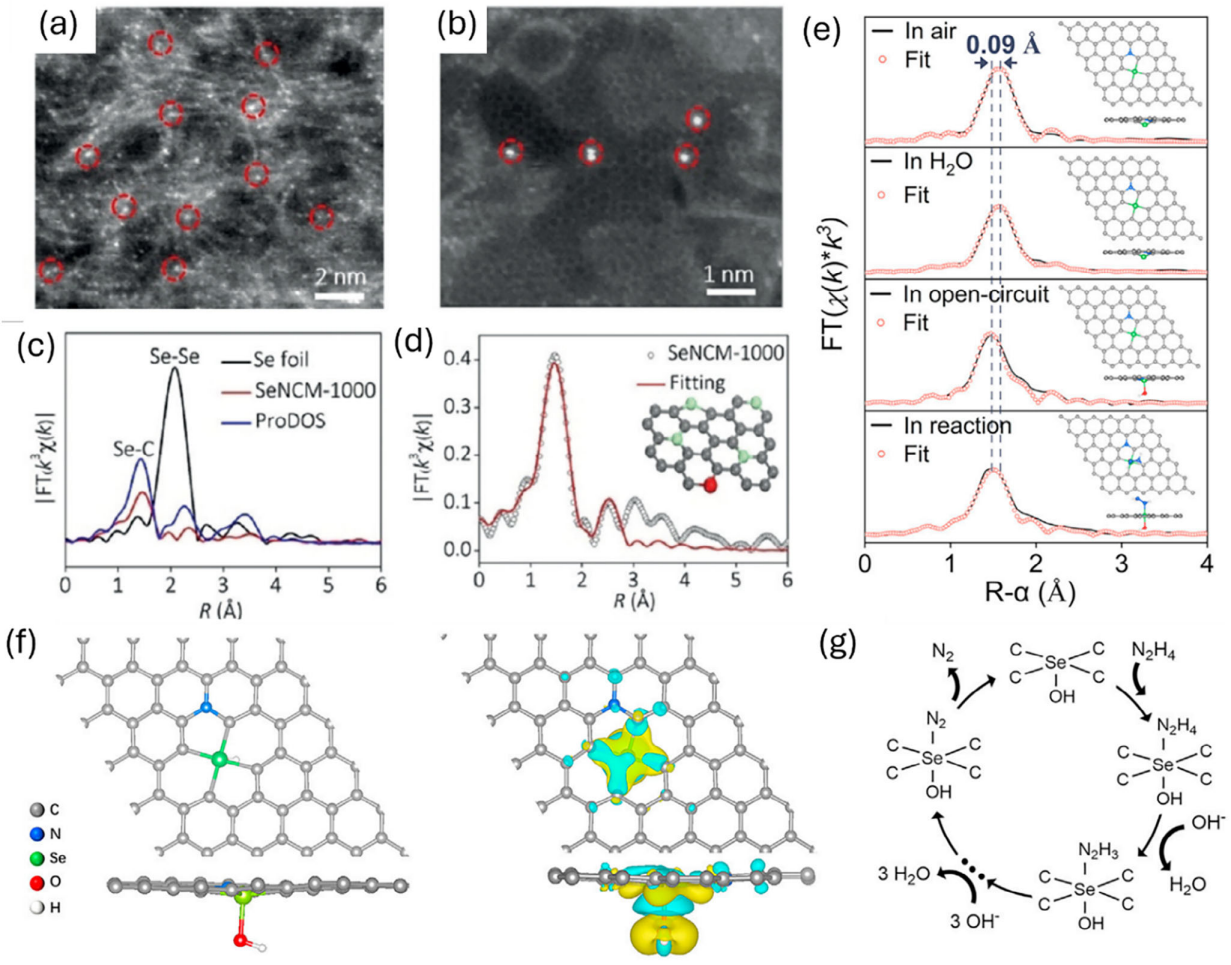
a,b) Scanning transmission electron microscopy (STEM) images of the Se‐NCMs. c) Selenium K‐edge Fourier transformed EXAFS spectra in the R space for Se‐NCMs compared with two reference samples. d) Fourier transformed EXAFS spectrum of Se‐NCMs along with the corresponding simulation curve. The inset shows the model structure of Se‐doped carbon membranes, where selenium is shown in red, carbon in grey, and nitrogen in green. Reproduced with permission.^[^ ^42^ ^]^ Copyright 2019, Wiley‐VCH. e) First‐shell fitting of EXAFS spectra. Inset in (e), corresponding geometric configurations. H, white; C, grey; N, blue; O, red; Se, green. f) Left: top and side views of the corresponding atomic configurations for OH–SeC_4_. Right: charge density difference analysis for OH–SeC_4_, with yellow regions indicating charge accumulation and cyan regions indicating charge depletion. g) Proposed hydrazine oxidation reaction (HzOR) mechanism on OH–SeC_4_. Reproduced with permission.^[^ ^43^ ^]^ Copyright 2024, Elsevier B.V.).

In sharp contrast, semi‐metallic elements can form comparably stable covalent bonds with carbon while exhibiting SAC‐like characteristics. By doping semi‐metal atoms, it is possible to address the stabilization challenge faced by traditionally carbon‐based SACs. However, the potential of these elements has been far less explored to date. In 2019, Wang et al. first prepared atomically dispersed Se atoms laden on N‐doped nanoporous carbon membranes (SeNCM) and demonstrated their chemical structure by a suite of insightful spectroscopy chracterizations.^[^ ^42^ ^]^ The isolated Se atoms were stabilized by carbon atoms in a ring structure (**Figure** **3**a,b). X‐ray absorption fine structure spectroscopy (XAFS) measurement demonstrated that Se atoms existed in a Se‐C covalent mode, and carried a positive charge and a valence state between 0 and + 4 (Figure 3c,d). Directly utilizing SeNCM as hydrazine fuel cell (HzFC) electrode, the performance tests showed that the open‐circuit potential, maximum current density and power density of the HzFC were 0.91 V, 605.3 mA cm^−2^ and 182.2 mW cm^−2^, respectively, competing with Pt/C based cell (0.92 V, 605.4 mA cm^−2^ and 170.9 mW cm^−2^). In situ X‐ray absorption spectroscopy (XAS) revealed that the pristine SeC_4_ site pre‐adsorbs an *OH ligand (Figure 3e), with the hydrazine oxidation reaction (HzOR) occurs on the opposite side of the OH–SeC_4_ (Figure 3f).^[^ ^43^ ^]^ Via theoretical calculations, the pre‐adsorbed *OH group withdraws electrons from the Se site, leading to a more positively charged Se and increased polarity of Se–C bonds, thereby enhancing surface reactivity toward HzOR (Figure 3g). It is worth highlighting that atomically dispersed Se atoms can form stable Se‐C covalent bonds, which endows the HzFC cells with long‐term operational stability. From this point of view, other semi‐metallic elements, including antimony (Sb), arsenic (As), germanium (Ge), and tellurium (Te), are feasible candidates for similar approaches. One uniqueness of semi‐metallic elements is that, unlike conventional transition metal elements (e.g., Pt^2^⁺ and M‐N₄ configurations), which exhibit high charge recombination rates due to their half‐filled d electrons. Semi‐metallic elements (e.g., Sb) possess a completely filled d¹⁰ electronic configuration.^[^ ^44^ ^]^ This configuration helps prevent the formation of an intermediate band in the band structure, thereby favoring efficient charge separation and the generation of reactive centers with a high density of electron holes.^[^ ^45^ ^]^ As a result, semi‐metallic elements show great promise as ideal candidates for photocatalytic applications. Thus, atomically dispersed semi‐metallic elements not only offer exceptional stability but also exhibit unique properties that can be leveraged in specific applications, surpassing the performance of conventional single‐atom catalysts (SACs). It is envisioned that semi‐metallic SACs could be pivotal in advancing the stability and efficiency of catalytic systems, enabling innovative applications in catalysis.

## 5 Emerging Preparation Precursors

In principle, anything with carbon atoms can be converted into carbon materials (even carp!^[^ ^46^ ^]^). Ideally, precursors rich in heteroatom are preferred to make heteroatoms doped functional carbon materials. For instance, carbon derived from renewable resources such as agricultural waste, wood, or algae presents an eco‐friendly alternative to fossil fuel‐based carbon materials.^[^ ^47^ ^]^ Through processes such as physical or chemical activation, biomass‐derived carbons can be engineered to achieve hierarchical porous structures comprising micropores, mesopores, and macropores, which facilitate adsorption, diffusion, and catalytic activity. Their applications span among energy storage, catalysis, environmental remediation, and more, positioning them as vital components in the advancement of green technologies and in addressing pressing environmental challenges.^[^ ^48^ ^]^ In addition, converting CO₂ into various carbon materials also offers sustainable alternatives to traditional carbon synthesis (Utilization of carbon dioxide as a carbon precursor: Review on mechanism and morphology).^[^ ^49^ ^]^ However, the carbonization of organic compounds, such as biomass, typically results in “poorly controlled” carbon materials, which are often amorphous and of low conductivity (≈10^2^–10^3^ S m^−1^). In a different manner, carbonization of ionic liquids (ILs) and their polymers poly(ionic liquid)s can produce carbon materials of a higher crystallinity. Many of these carbon materials are termed “noble carbons” due to their ability to withstand high temperatures in the presence of oxygen over extended periods.^[^ ^50^ ^]^ Direct pyrolysis of MOFs (or COF) has recently stimulated much interest because their intrinsic micropores could be partially retained during carbonization.^[^ ^51^ ^]^ More importantly, the metal ion centres after a thermos‐reduction step can evaporate (i.e., Zn) to leave behind porous structures or to in situ corporate metal sites into carbon materials. From a practical application standpoint, the pyrolysis of organic frameworks such as MOFs or COFs typically yields a low carbon output, and the preparation of the resulting carbons can be quite costly. This presents a challenge in scaling up these materials for real‐life uses.

Equally important is the porosity of carbon materials, which is essential for exposing active sites on the carbon matrix and maximizing the performance of heteroatom‐doped carbons. Utilizing different carbonaceous precursors can impart desired porosity to the carbon. Previous reviews commendably discussed on how to create porous structures in carbon materials, which is beyond this minireview.^[^ ^7^, ^52^ ^]^

## 6 Carbonization Techniques

**Figure 4:**
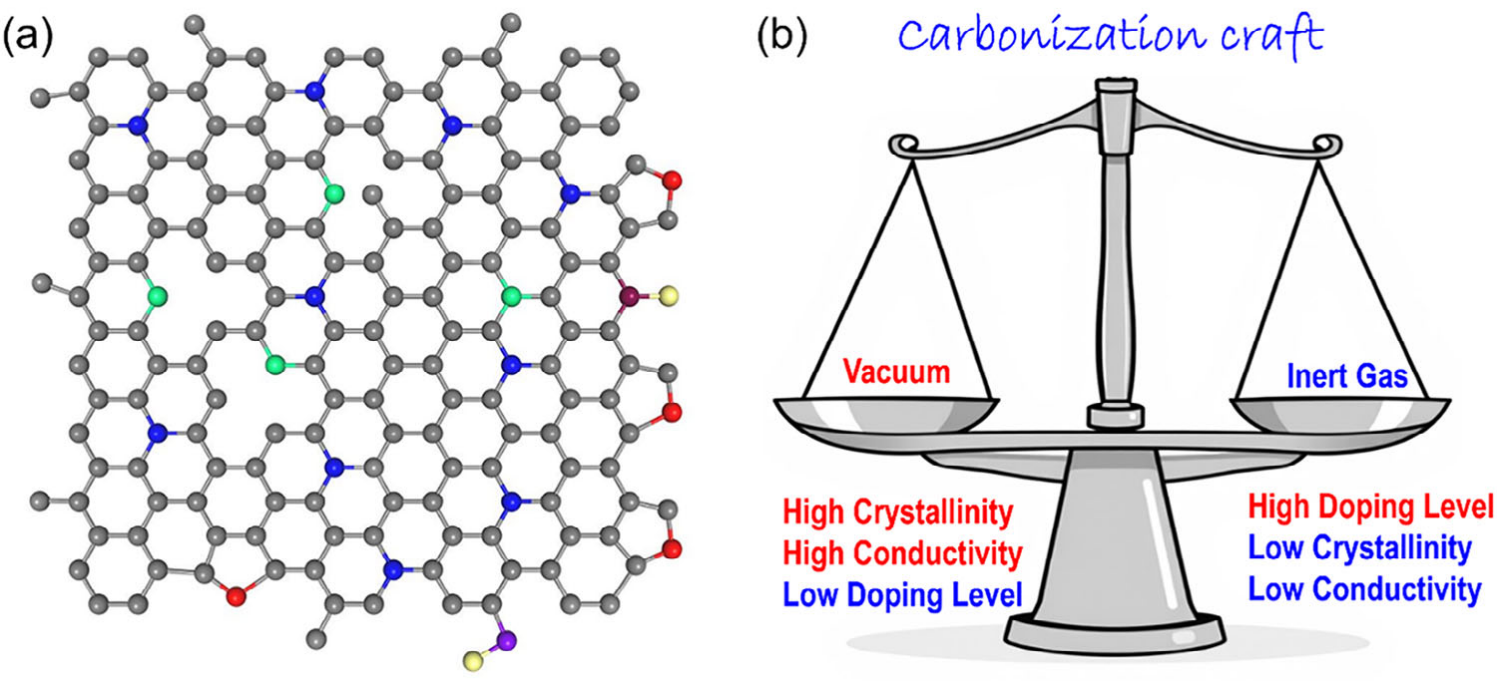
Schematic illustration of (a) heteroatoms doped carbon and (b) comparison of carbonization techniques under inert gas and under vacuum conditions.

No doubt that carbonization conditions will determine to a large extent the physicochemical properties of carbon materials. For instance, in the case of F‐doped carbons, pyrolysis at temperatures between 400 to 600 °C can favor a relatively high content of more ionic C─F bonds. It is believed that the content of more ionic C─F bonds is crucial for the overall ORR activity.^[^ ^26a^ ^]^ However, increasing the temperature would break down these C─F bonds, reducing the overall F content. This process typically enhances the electrical conductivity of carbon materials, which could also be beneficious for electrochemical application. In this regard, designing a material that perfectly balances these trade‐offs can be quite complex, as each adjustment impacts various properties differently. Similar, carbonization in an inert atmosphere such as nitrogen (N₂) and argon (Ar) has been a default method to produce carbon materials. However, the rapid degradation kinetics during vacuum carbonization can favor more disordered carbon structures than those formed under typical inert conditions. The swift removal of some heteroatoms during vacuum carbonization adds extra disorder to carbon matrix.^[^ ^53^ ^]^ This process often retains fewer heteroatoms in carbon products but increases electrical conductivity (up to ≈10⁵ S m^−1^), while carbonization under inert retains more heteroatoms but may reduce crystallinity (≈10⁴ S m^−1^).^[^ ^54^ ^]^ Here, a trade‐off between heteroatom content, crystallinity, and conductivity must be carefully considered and balanced for maximum performance for specific applications. This balance is key to tailoring carbon materials to meet the diverse demands (**Figure** **4**). It is also worth highlighting that hydrothermal carbonization is a sustainable, versatile, and powerful process for transforming biomass‐wastes into functional carbon materials.^[^ ^55^ ^]^ Biomass precursors such as sugar are often used to afford carbon spheres. Silica templates and salts can also be used to introduce porosity. These carbon materials are rich in heteroatoms and can be used as metal support for different catalytic reactions.

## 7 Conclusion and Future Perspective

Chemical dopants offer valuable opportunities for upgrading carbon materials. This process extends beyond conventional heteroatoms and includes halogens, semi‐metallic elements, and even metallic elements in the form of single atom catalysts (SACs). These innovations present promising prospects and unique properties to advance traditional doping methods, particularly important to the fields of energy storage and electrochemical catalysis. It opens up new possibilities for re‐design catalyst performance, potentially mitigating the activity‐stability trade‐off commonly observed in SACs. In addition, careful consideration in carbonaceous precursors and optimizing carbonization craft can develop carbon materials with improved structural integrity, desirable physiochemical properties and enhanced functionalities for specific application.

The practical application of engineered carbon materials faces several challenges: i) Achieving precise control over the heteroatom configuration of each dopant in the final pyrolyzed carbon materials is a complex task. The nature and geometry of the active sites can significantly influence reaction rates, mechanisms and selectivity. In this regard, pyrolyzed carbon materials with structural and compositional inconsistencies could result in undesirable side reactions and unpredictable performances. Efforts are currently being made to achieve exclusive heteroatom configurations in doped carbon materials; however, this has yet to be systematically demonstrated in the case of halogen‐doped or multi‐doped carbons. To address these challenges, future research should prioritize the nanoengineering technologies grounded in fundamental reaction mechanisms. ii) Utilizing artificial intelligence (AI) is revolutionizing the development of advanced carbon materials, with a focus on accelerating discovery, design, and optimization processes.^[^ ^56^ ^]^ However, the current datasets for materials are incomplete, noisy, or biased toward successful experiments. In this regard, it is necessary to standardize experiments procedures and outcomes to integrate datasets for machine learning training. iii) To scale‐up production of engineered carbon materials is essential. It encompasses the cost of carbonaceous precursors, optimization of the carbonization process, and maintaining the desired functionalities during scale‐up. Addressing these issues will be critical in fulfilling full potential of chemically doped carbon materials in various applications.

## Conflict of Interest

The authors declare no conflict of interest.
